# Exploring genetic diversity and variation of *Ovar-DRB1* gene in Sudan Desert Sheep using targeted next-generation sequencing

**DOI:** 10.1186/s12864-024-10053-3

**Published:** 2024-02-08

**Authors:** Bashir Salim, Ryo Nakao, Elisha Chatanga, Olivia Marcuzzi, Muna Ahmed Eissawi, Faisal Almathen, Olivier Hanotte, Guillermo Giovambattista

**Affiliations:** 1 Department of Parasitology, Faculty of Veterinary Medicine, University of Khartoum, Khartoum-North, Sudan; 2 Camel Research Center, King Faisal University, Al-Hasa, Saudi Arabia; 3 Laboratory of Parasitology, Department of Disease Control, Faculty of Veterinary Medicine, Hokkaido University, Sapporo, Japan; 4 Facultad de Ciencias Veterinarias, Universidad Nacional de La Plata, IGEVET – Instituto de Genética Veterinaria (UNLP‐CONICET LA PLATA), La Plata, Argentina; 5 Animal Resources Research Corporation, Khartoum Sudan, Sudan; 6 Department of Veterinary Public Health and Animal Husbandry, College of Veterinary Medicine, King Faisal University, Al-Ahsa, Saudi Arabia; 7 Cells, Organisms and Molecular Genetics, School of Life Sciences, University of Nottingham, Nottingham, UK; 8 International Livestock Research Institute, Addis Ababa, Ethiopia

**Keywords:** *Ovar-DRB1* gene, Next-generation sequencing, Genetic diversity, Sudan Desert Sheep

## Abstract

**Introduction:**

The *Ovar-DRB1* gene, a crucial element of the Major Histocompatibility Complex (MHC) Class II region, initiates adaptive immunity by presenting antigens to T-cells. Genetic diversity in sheep, particularly in MHC Class II genes like *Ovar-DRB1*, directly influences the specturm of presented antigens impacting immune responses and disease susceptability. Understanding the allelic diversity of *Ovar-DRB1* gene in Sudan Desert Sheep (SDS) is essential for uncovering the genetic basis of immune responses and disease resistance, given the the breeds significance in Sudan's unique environment.

**Methods:**

Utilizing Targeted Next-Generation Sequencing (NGS) we explore allelic diversity in *Ovar-DRB1* gene within SDS. Successfully ampliying and and sequencing the second exon of this gene in 288 SDS samples representing six breeds provided a comprehensive allelic profile, enabling a detalied examination of the gene's genetic makeup.

**Results:**

We identifed forty-six alleles, including four previously unreported, enrichness the genetic diversity of SDS breeds. These alleles exhibiting non-uniform distribution, varying frequencies across breeds, indicating a breed-specific genetic landscape. Certain alleles, known and novel, show higher frequencies in specific populations, suggesting potential associations with adaptive immune responses. Identifying these alleles sets the stage for investigating their functional roles and implications for disease resistance. Genetic differentiation among SDS breeds, as indicated by *F*_*ST*_ values and clustering analyses, highlights a unique genetic makeup shaped by geographic and historical factors. These differentiation patterns among SDS breeds have broader implications for breed conservation and targeted breeding to enhance disease resistance in specific populations.

**Conclusion:**

This study unveils *Ovar-DRB1* gene allelic diversity in SDS breeds through targeted NGS and genetic analyses, revealing new alleles that underscore the breeds’ unique genetic profile. Insights into the genetic factors governing immune responses and disease resistance emerge, promising for optimization of breeding strategies for enhanced livestock health in Sudan’s unique environment.

**Supplementary Information:**

The online version contains supplementary material available at 10.1186/s12864-024-10053-3.

## Introduction

Major Histocompatibility Complex (MHC) genes play a crucial role in immune responses and disease resistance in various species, including sheep. Among the MHC genes, *Ovar-DRB1* has garnered significant attention due to its involvement in antigen presentation and recognition [1, 2].Understanding the genetic diversity and variation within the *Ovar-DRB1* gene is essential for breeding programs and conservation efforts in sheep populations [3].

Sudan Desert Sheep (SDS) are a unique breed adapted to the harsh environmental conditions of the Sudanese desert. These sheep possess remarkable traits, such as heat tolerance, drought resistance, infectious diseases tolerance and adaptation to sparse grazing resources, making them well-suited for arid regions. However, the genetic characteristics and diversity of the *Ovar-DRB1* gene in SDS remain largely unexplored [4, 5]**.**

To fill this knowledge gap, we conducted a comprehensive study using targeted Next Generation Sequencing (NGS) technology to genotype the *Ovar-DRB1* gene in SDS. Targeted NGS allows for cost-effective and efficient genotyping of specific genomic regions, making it a powerful tool for investigating genetic diversity in targeted genes.

In recent studies, the genetic diversity of native sheep breeds reared in Turkey and Algeria has been investigated [6, 7]. These studies aimed to explore the impact of polymorphisms in the MHC gene region, which has the potential to influence future animal breeding strategies. In line with these efforts, [8] conducted Sanger sequencing of the highly polymorphic Exon 2 of the ovine DRB1 gene. Their study focused on six indigenous Turkish sheep breeds and two crossbreeds, aiming to uncover the diversity within this gene. Additionally, [9] described nine novel full-length *Ovar-DRB1* sequences and introduced an improved direct-sequencing method for analyzing the entire exon 2 region of the *Ovar-DRB1* gene, incorporating previously unknown intronic sequences. These investigations enhance our understanding of MHC gene polymorphisms and their potential significance for the development of f forthcoming breeding initiatives in animal populations.

In this paper, we present the results of our genotyping analysis of the *Ovar-DRB1* gene in Sudan Desert Sheep using targeted NGS. We collected DNA samples from a representative population of Sudan Desert Sheep and employed a targeted sequencing approach to specifically capture and sequence the *Ovar-DRB1* gene regions of interest. Through this targeted NGS approach, we obtained high-quality DNA sequence data for the Ovar-DRB1 gene in Sudan Desert Sheep. Our analysis focused on identifying genetic variants, assessing allele frequencies, and exploring the population structure and diversity within the Sudan Desert Sheep population [10, 11].

By elucidating the genetic diversity and variation within the *Ovar-DRB1* gene, our study provides valuable insights into the adaptive potential and immune responses of Sudan Desert Sheep. This information is crucial for implementing effective breeding strategies, improving disease resistance, and ensuring the long-term health and resilience of this unique breed. Furthermore, the results of our study contribute to the broader understanding of MHC genetics in sheep populations. The targeted NGS approach employed in this research can serve as a valuable tool for future studies investigating the MHC diversity and functional significance in other sheep breeds or related species [12].

In conclusion, our study demonstrates the utility of targeted NGS for genotyping the *Ovar-DRB1* gene in Sudan Desert Sheep, shedding light on the genetic diversity and variation within this important MHC gene. The findings of this research have practical implications for breeding programs, conservation efforts, and the broader understanding of MHC genetics in sheep populations.

## Materials and methods

### Sample population collection and genomic DNA extraction

**Fig. 1:**
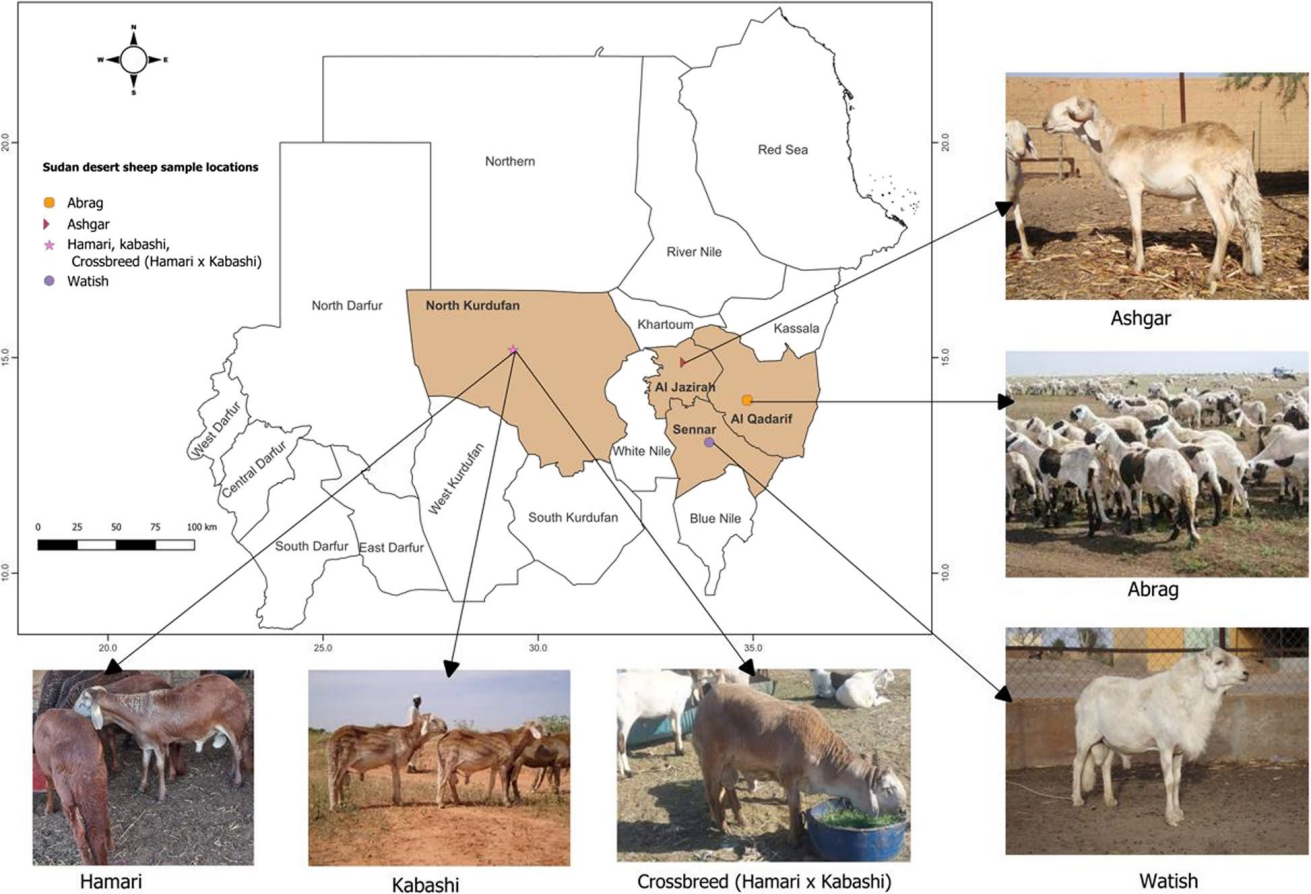
Geographic Distribution of Sudan Desert Sheep (SDG) Breeds and Blood Sample Collection Sites: This map displays the geographic locations where blood samples were collected from various Sudan Desert Sheep (SDG) breeds across four states. The scale is in kilometers

A total of 288 blood samples were collected from various Sudan desert sheep (SDS) breeds, including Abrag (AB; *n* = 37), Ashgar (AS; *n* = 44), Buze´e (B; *n* = 23), Hamari (H; *n* = 72), Kabashi (K, *n* = 45), and Watish (W; *n* = 38). The samples were obtained from regions located in Al Gadarif, Al Jazirah, Sennar, and North Kurdufan States as illustrated in the map in (Fig. 1), showcasing the geographical diversity of the sampled Sudan desert sheep breeds. Prior consent was obtained from a shepherd, and precautions were taken to avoid sampling closely related individuals. Genomic DNA extraction was performed using the DNeasy® Blood and Tissue Kit (Qiagen, Germany) according to the manufacturer's instructions. This extraction method ensures the isolation of high-quality DNA suitable for downstream analyses.

### Ethical approval

The study protocol was reviewed and approved by the Faculty of Veterinary Medicine, University of Khartoum. The research was conducted in accordance with their guidelines for sampling domestic animals in Sudan, ensuring ethical compliance in the collection and use of the samples.

### PCR amplification and sequencing

For typing *Ovar-DRB1* breeds, we implemented a method based on targeted Next-Generation Sequencing. First, the second exón of this gene were amplified using the primers Illumina_330: TCGTCGGCAGCGTCAGATGTGTATAAGAGACAGNNNNNNATTAGCCTCYCCCCAGGAGKC and Illumina_329: GTCTCGTGGGCTCGGAGATGTGTATAAGAGACAGNNNNNNCACCCCCGCGCTCACCTCGCCGC based on a previous study amplifying the region using conventional PCR [13]. The PCR reaction mixture contained 12.5 μl of 2X KAPA HiFi HotStart ReadyMix (Kapa Bio-Systems, Wilmington, MA, USA), 200 nM of each primer and 2.5 μl of the genomic DNA. The reaction was performed at 95 °C for 3 min, followed by 35 cycles of 95 °C for 30 s, 60 °C for 30 s, 72 °C for 30 s and a final extension at 72 °C for 5 min. PCR amplicons were visualized by electrophoresis on 1.5% agarose gel stained with Gel-RedTM (Biotium, Hayward, CA). Illumina sequencing libraries were prepared by purifying the amplicons using AMPure XP (Beckman Coulter Life Sciences, USA) and sequencing adapters and index sequences were added using the Nextera XT Index Kit (Illumina, CA, USA). The sequencing run was conducted with a MiSeq Reagent Kit v3 (600 cycles) on an Illumina MiSeq device according to the manufacturer’s instructions.

#### Sequence data analysis and *O*var*-DRB1* allele genotyping

The quality of obtained Fastq files was evaluated using FastQC software [14] FastQ files were analyzed following the best practice recommended by GATK Germline short variant discovery (SNPs + Indels) (https://gatk.broadinstitute.org/hc/en-us/articles/360035535932-Germline-short-variant-discovery-SNPs-Indels-). Briefly, raw reads were mapped to the ARS-UI_Ramb V2.0 Assembly version of the ovine (*Ovis aries*) genome (https://www.ncbi.nlm.nih.gov/assembly/GCF_016772045.1/) using BWA-MEM aligner [15] to generate BAM and SAM files. Variants (SNPs and INDELs) per sample were called using HaplotypeCaller to generate GVCF files and consolidated using the joint-call cohort to generate the two VCF files, one for SNPs and other for INDELs. Then, variants were filtered, refined, and annotated to obtain the final VCF file. The SnpEff version 4.3.1 software (https://pcingola.github.io/SnpEff/) Variants and haplotypes were visualized using the IGV software (reference). Heterozygous sites were confirmed when both alleles have balanced numbers of reads. Obtained DNA sequences were compared with previously reported *Ovar-DRB1* alleles in the IPD-MHC database to assign the genotypes. Sequences were submitted to the GenBank (https://www.ncbi.nlm.nih.gov/genbank/) under accession numbers from OR488718-OR488752, OR488756, OR488764, OR488770, and OR636395.

### Statistical analyses

#### Genetic diversity at allele level

Allele frequencies and the number of alleles (n_a_) were obtained by direct counting. The distribution of alleles across breeds was analyzed and visualized by a Venn plot created using the R package ‘VennDiagram’ (http://cran.r-project.org/; [16]. The observed (h_O_) and unbiased expected (h_E_) heterozygosity of the *Ovar-DRB1* locus were estimated according to [17] using the Arlequin 3.5 software for population genetic analyses [18]. F_IS_ statistics for each breed were calculated using the Exact Test proposed by [19] and implemented in Genepop 4.7 software [20] to evaluate deviation from Hardy–Weinberg equilibrium (HWE). The Ewens–Watterson–Slatkin Exact Test of neutrality was performed using the method described by [21] and implemented in the Arlequin 3.5 program.

#### Genetic structure of the Sudan Desert Sheep

Genetic structure and genetic differentiation among sheep breeds were assessed using Wright’s FST statistics**,** calculated using the variance-based method of [19]. This parameter was estimated using Arlequin 3.5 and Genepop 4.7 software. The fixation index (F_ST_) values were represented graphically using the pairFstMatrix.r function implemented in the R statistical environment. To condense the genetic variation at the *Ovar-DRB1* locus, allele frequencies were used to perform a Principal Component Analysis (PCA) according to [22] implemented in Past software [23]. Nei’s 1972 standard genetic distances [24] Ds and DA [25] were calculated from allele frequencies and were used to perform cluster analysis using the unweighted pair-group method with arithmetic mean (UPGMA;[26] and the neighbor-joining algorithm (NJ; [27]. Confidence intervals for the groupings were estimated by bootstrap resampling of the data using 1000 replicates. Population genetic distances and trees were computed and visualized using the POPTREE2 [28].

#### Genetic diversity at sequence level

Nucleotide diversity (π) and pairwise differences in nucleotide substitutions between alleles (NPD) within each sheep breed were calculated using Arlequin 3.5. The mean number of nonsynonymous (dN), and synonymous (dS) nucleotide substitutions per site calculated as an average over all sequence pairs were estimated within each group using the modified Nei-Gojobori model [29] and Jukes–Cantor’s formula implemented in the software MEGA X [30].

The possibility that certain codon sites are under diversifying selection within each native Sudan breed was investigated using the Bayesian method implemented using OmegaMap [31]. This method incorporates intragenic recombination and does not assume a known fixed genealogy, so that recombination does not inflate the false detection rate of positive sites. The *Ovar-DRB1* allele tree was conducted by using the Maximum Likelihood method and Tamura-Nei model [32] implemented into the MEGA X software. Ovar-DQB1*01:01:01 allele was included as an outgroup.The bootstrap consensus trees were inferred from 1000 replicates. These trees were constructed based on the complete second exon DNA sequences or only the codons that codify the antigen-binding site (ABS) amino acid motifs.

## Results

### Targeted NGS genotyping method

In the present work, a targeted NGS method was implemented to genotype the *Ovar-DRB1* gene. The second exon of this gene was amplified from 288 animals belonging to SDS, and the PCR products were pooled and sequencing in a single run. This analysis allowed us to obtain a full coverage of the second exon (270 bp) that includes the β1 domain with an average depth of 32.61 ± 18.29 in 259 animals, failing only 29 samples. Overlapping of forward and reverse reads allowed to determine the allele phases.

### Distribution of *Ovar-DRB1* alleles in selected native Sudan Desert sheep breeds

**Fig. 2:**
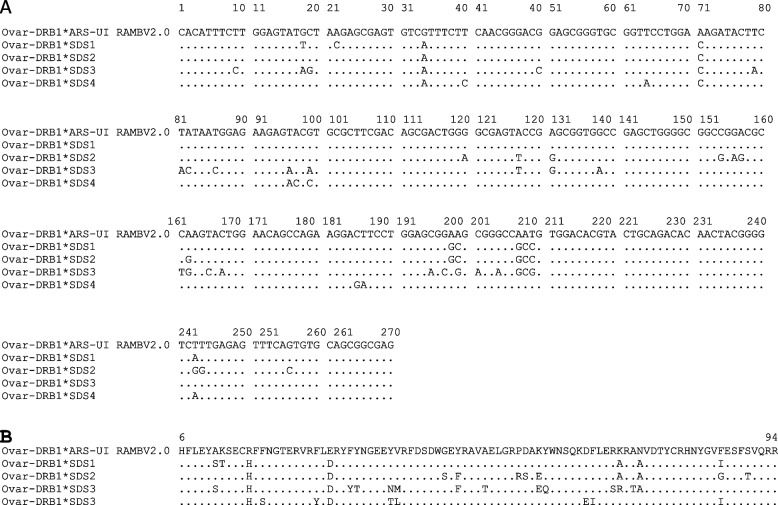
Alignment of the nucleotide (**a**) and the predicted amino acid (**b**) sequences of the β1 domain encoded by four new *Ovar-DRB1* alleles (accession numbers: OR488764 for Ovar-DRB1*SDS1, OR488756 for Ovar-DRB1*SDS2, OR488770 for Ovar-DRB1*SDS3, OR636395 for Ovar-DRB1*SDS24) derived from 259 Sudan Desert Sheep (37 animals of the Abrag, 44 of the Ashgar, 23 of the Buze´e, 72 of the Hamari, 45 of the Kabashi, and 38 of the Watish). Numbering refers to amino acid positions in the mature protein. Nucleotide and amino acid residues identical to those encoded by the *Ovar-DRB1* sequence from the the ARS-UI_Ramb V2.0 Assembly version of the ovine (*Ovis aries*) genome are indicated by dots (https://www.ncbi.nlm.nih.gov/assembly/GCF_016772045.1/)

**Fig. 3:**
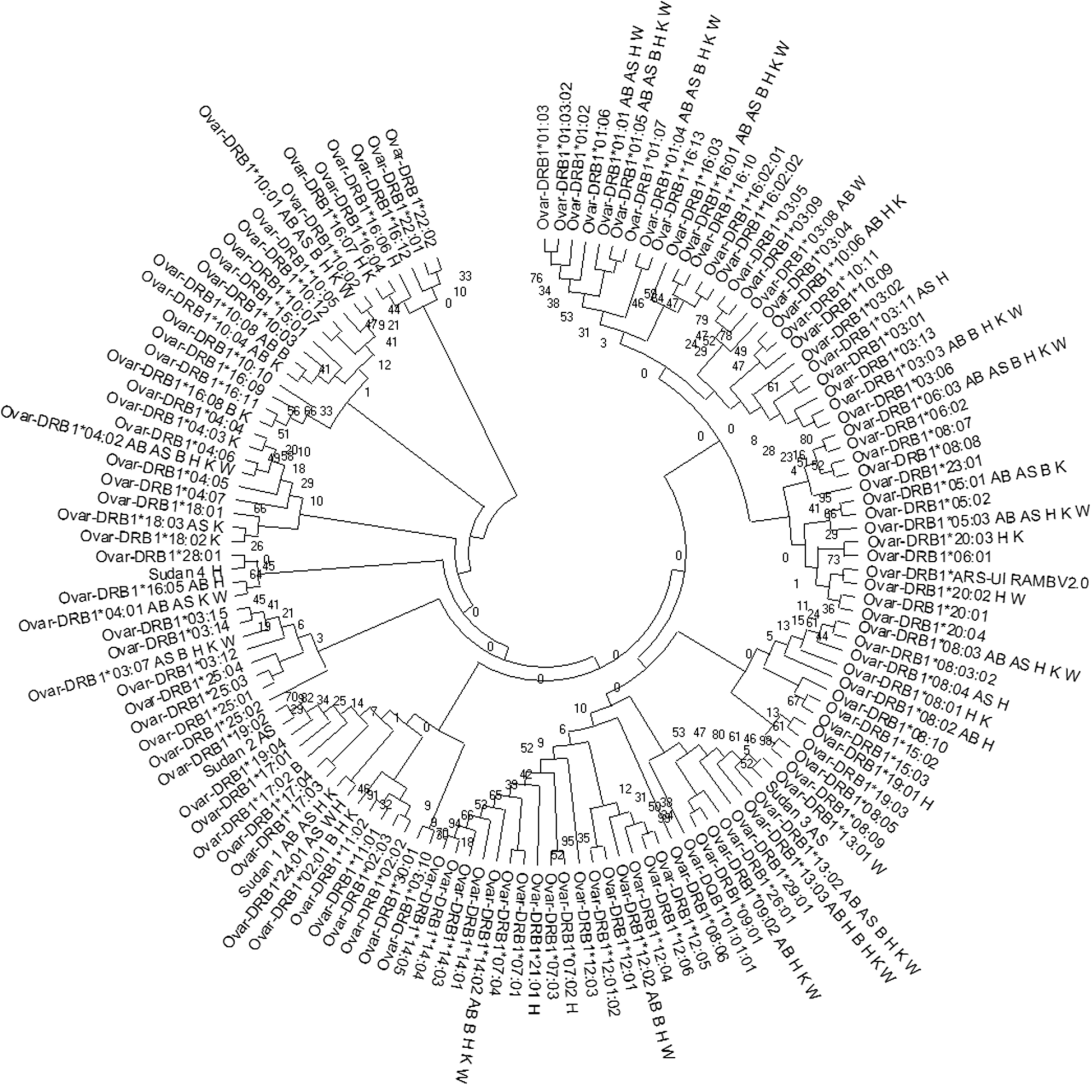
Neighbor-joining (NJ) tree constructed from the 270 bp nucleotide sequence that includes the β1 domain encoded by all reported *Ovar-DRB1* alleles and the twenty-six new ones (From Ovar-DRB3*Sudan 1 to Ovar-DRB1*Sudan 26) detected in the Sudan desert sheep breeds. Numbers are bootstrap percentages that support each node. Bootstrapping was carried up with 1000 replicates to assess the reliability of individual branches. AB = Abrag, AS = Ashgar, B = Buze´e, H = Hamari, K = Kabashi, and W = Watish

Targeted NGS genotyping allowed us to identify forty-six *Ovar-DRB1* alleles (42 previously reported variants and four new alleles; Table 1 and Fig. 2) in the SDS breeds. The number of alleles (n_a_) varied from 17 in B to 33 in H (Tables 1 and 2). The new *Ovar*-DRB1*Sudan1 was detected in four SDS breeds, whereas the remaining three new alleles were present in two heterozygous animals in Ashgar breed (*Ovar*-DRB1*Sudan2 and *Sudan3) and one animal in Hamari (*Ovar*-DRB1*Sudan4). Nucleotide and predicted amino acid sequences of the four new alleles are shown in Fig. 2 and compared with the *Ovar-DRB1* DNA sequence of the ARS-UI_Ramb V2.0 Assembly. Figure 3 showed that the variants detected in Sudan desert sheep breeds were interspersed among the various clusters of the *Ovar-DRB1* tree, including all the previously reported alleles and the four new alleles. A similar result was observed when the *Ovar-DRB1* tree was inferred using codons corresponding to the ABS (Fig. S1).

**Table 1:** *Ovar-DRB1* gene frequencies estimated for Sudan Desert Sheep breeds. Abrag (AB; *n* = 37), Ashgar (AS; *n* = 44), Buze´e (B; *n* = 23), Hamari (H; *n* = 72), Kabashi (K, *n* = 45), and Watish (W; *n* = 38)

Ovar-DRB1 Alleles	AB	AS	B	H	K	W
Ovar-DRB1*01:01	2,70	4,55		1,39		2,63
Ovar-DRB1*01:04	**5,41**	**6,82**	**6,52**	0,69	4,44	3,95
Ovar-DRB1*01:05	1,35	1,14	2,17	2,08	3,33	1,32
Ovar-DRB1*02:01			4,35	2,78	3,33	
Ovar-DRB1*03:03	1,35		**10,87**	4,17	**6,67**	1,32
Ovar-DRB1*03:07		2,27	**10,87**	3,47	2,22	1,32
Ovar-DRB1*03:08	1,35					1,32
Ovar-DRB1*03:11		3,41		1,39		
Ovar-DRB1*04:01	1,35	1,14			**6,67**	3,95
Ovar-DRB1*04:02	**9,46**	**5,68**	**10,87**	**13,19**	**10,00**	**15,79**
Ovar-DRB1*05:01	1,35	2,27	2,17		2,22	
Ovar-DRB1*05:03	**9,46**	1,14		2,08	1,11	**6,58**
Ovar-DRB1*06:03	**10,81**	**18,18**	**6,52**	**7,64**	**12,22**	**10,53**
Ovar-DRB1*07:02				0,69		
Ovar-DRB1*08:01				2,08	1,11	
Ovar-DRB1*08:02	2,70			0,69		
Ovar-DRB1*08:03	1,35	3,41		1,39	2,22	1,32
Ovar-DRB1*08:04		1,14		0,69		
Ovar-DRB1*09:02	1,35			0,69	3,33	5,26
Ovar-DRB1*10:01	**13,51**	**27,27**	**6,52**	**17,36**	**17,78**	**7,89**
Ovar-DRB1*10:04	2,70				1,11	
Ovar-DRB1*10:06	1,35			0,69	1,11	
Ovar-DRB1*10:08	1,35		2,17			
Ovar-DRB1*12:02	**5,41**		**6,52**	2,08		**6,58**
Ovar-DRB1*13:01						2,63
Ovar-DRB1*13:02	**8,11**	**7,95**	**6,52**	**6,94**	2,22	**11,84**
Ovar-DRB1*13:03	**6,76**	1,14	**6,52**	4,86	1,11	**9,21**
Ovar-DRB1*14:02	1,35		**8,70**	0,69	1,11	2,63
Ovar-DRB1*14:03					1,11	
Ovar-DRB1*16:01	4,05	3,41	2,17	2,78	1,11	1,32
Ovar-DRB1*16:05	2,70			0,69		
Ovar-DRB1*16:06				0,69		
Ovar-DRB1*16:07				0,69	1,11	
Ovar-DRB1*16:08			4,35		1,11	
Ovar-DRB1*17:02			2,17			
Ovar-DRB1*18:02					2,22	
Ovar-DRB1*18:03		1,14			2,22	
Ovar-DRB1*19:01				0,69		
Ovar-DRB1*20:02				3,47		1,32
Ovar-DRB1*20:03				2,08	2,22	
Ovar-DRB1*21:01				0,69		
Ovar-DRB1*24:01		1,14		0,69		1,32
Ovar-DRB1*Sudan1	2,70	4,55		**9,03**	**5,56**	
Ovar-DRB1*Sudan2		1,14				
Ovar-DRB1*Sudan3		1,14				
Ovar-DRB1*Sudan4				0,69		

*N* = number of animals analysed; frequent alleles in each ecotype are indicated in bold (> 5%); and novel alleles identified in this study are indicated in bold and underlined

Ovar-DRB1 alleles were named following the recommended IPD-MHC nomenclature (species and locus designation* allelic family: coding change within the allelic family; IPD-MHC Database (ebi.ac.uk))

**Table 2:** Number of alleles (na) observed (h_O_) and expected heterozygosity (h_E_), F_IS_ index and Slatkin´s exact neutrality test estimated for *Ovar-DRB1* gene in Abrag (AB), Ashgar (AS), Buze´e (B), Hamari (H), Kabashi (K), and Watish (W) Sudan Desert Sheep breeds. *N* = sample size

Breed	N	na	h_O_	H_E_	F_IS_ (*p* value)	Slatkin's Exact test *P*-value
AB	37	24	0.92	0.94	0.024 (0.21)	0.342
AS	44	21	0.82	0.88	0.069 (0.74)	0.748
B	23	17	1.00	0.95	-0.06 (0.53)	**0.014**
H	72	33	0.88	0.93	0.06 (0.22)	0.707
K	45	27	0.89	0.93	0.05 (0.77)	0.485
W	38	21	0.87	0.93	0.07 (0.13)	0.267

**Fig. 4:**
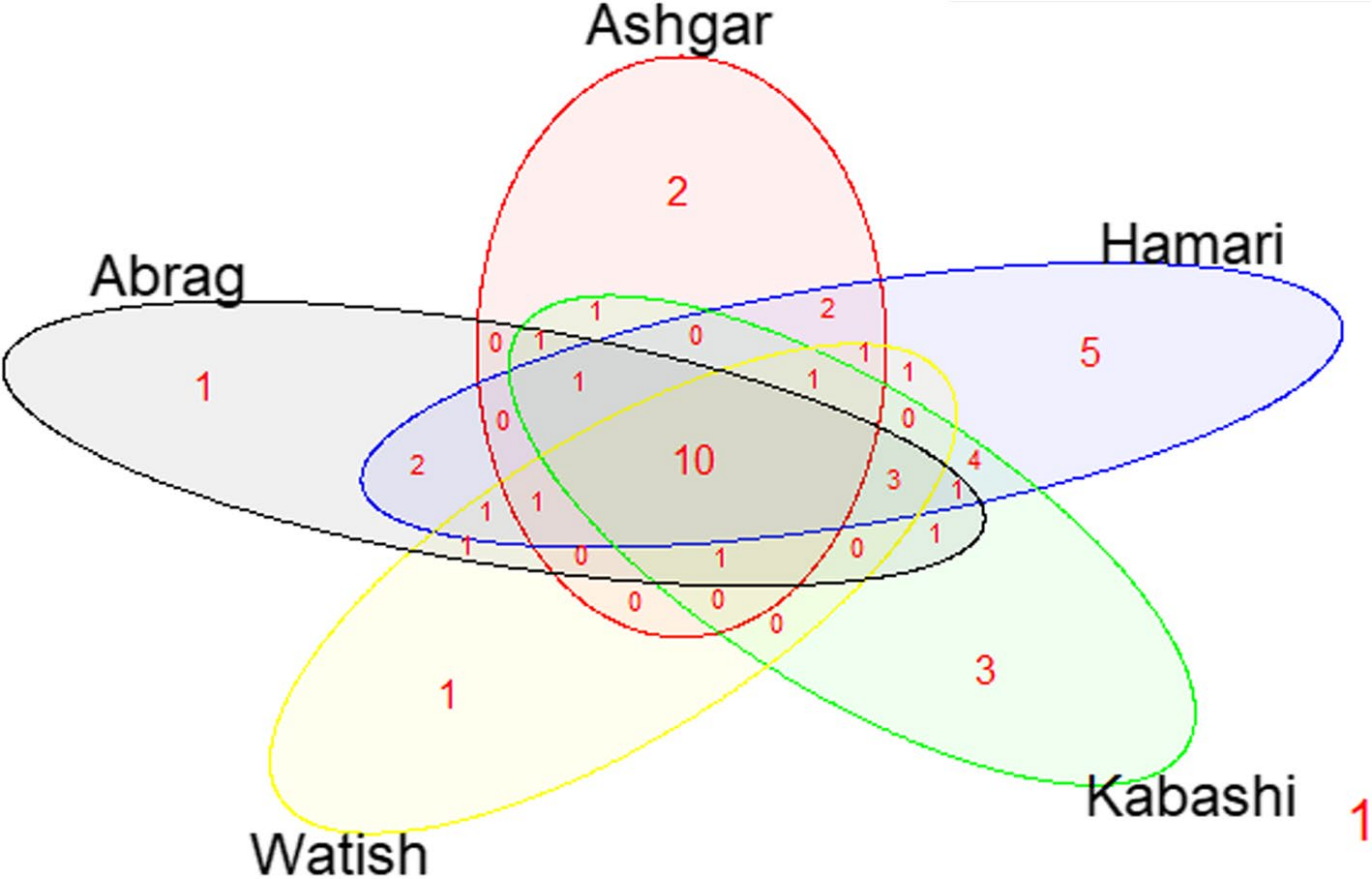
Venn plot of *Ovar-DRB1* alleles shared by Sudan Desert Sheep breeds: Abrag, Ashgar, Hamari, Kabashi, and Watish breeds

A Venn diagram was constructed using data obtained in this study to analyze and illustrate the distribution of the forty-six *Ovar-DRB1* alleles detected in the SDS sample analyzed. As shown in Fig. 4, this analysis revealed that out of the forty-six alleles identified in SDS, only ten were identified in all breeds, twelve variants were unique for one population, whereas the remaining were detected in from two to four breeds. In addition, variant Ovar-DRB1*17:02 only detected the Buze´e crossbreed population.

As shown in Fig. S2, the SDS breeds have an even gene frequency distribution, with a high number of alleles with low frequency. Between five to eight alleles appeared with frequencies of > 5% within each population. These common alleles accounted for a cumulative gene frequencies that range from 54.20% in H to 80.43% in K; three (Ovar-DRB1*04:02, *06:03, and *10:01) of which were common in all SDS breeds, one (Ovar-DRB1*13:02) was detected in four out of the five populations, whereas thirty-four variants presented gene frequencies lower than 5% (Table 1, Fig. S3).

### Nucleotide and amino acid diversity in the *Ovar-DRB1* alleles found in Sudanese Desert Sheep breeds

Genetic diversity at the DNA and amino acid levels was evaluated using methods that.

compare the average amino acid and nucleotide substitutions for every pair of alleles within the breeds studied. Table 3 summarized the results obtained with these methods. The π values exceeded 0.055 (πrange = 0.055 – 0.074) and the mean number of pairwise difference values exceeded 16.73 (NPD range = 14.95 – 19.95) within Sudan Desert Sheep populations. Regarding amino acid diversity, the average dN and dS substitutions in these sheep breeds were calculated across the entire *Ovar-DRB1* exon 2 and the ABS. The dN/dS ratio observed in SDS ranged from 0.026 to 0.043 for the entire second exon and from 0.212 to 0.236 when only ABS were considered.

**Table 3:** Nucleotide diversity (π), mean number of pairwise differences (NPD) and mean number of non-synonymous (d_n_) and synonymous (d_s_) nucleotide substitutions per site estimated for the second exon of the *Ovar-DRB1* gene within each Sudan Desert Sheep Breeds: Abrag (AB), Ashgar (AS), Hamari (H), Buze´e (B), Kabashi (K), and Watish (W)

Ecotypes	π	NPD	Total			ABS		
			d_s_	d_n_	*d*_*s*_*—d*_*n*_	d_s_	d_n_	*d*_*s*_*—d*_*n*_
AB	0.064	17.19	0.034	0.077	-0.043	0.046	0.275	-0.229
AS	0.055	14.95	0.040	0.082	-0.042	0.039	0.264	-0.225
B	0.064	17.20	0.041	0.081	-0.041	0.041	0.268	-0.228
H	0.058	15.65	0.045	0.079	-0.033	0.051	0.263	-0.212
K	0.057	15.45	0.042	0.082	-0.039	0.046	0.282	-0.236
W	0.074	19.95	0.062	0.087	-0.026	0.060	0.286	-0.226

### Gene diversity, Hardy–Weinberg Equilibrium (HWE), and neutrality testing of BoLA-DRB3 variants found in Sudanese cattle breeds

**Fig. 5:**
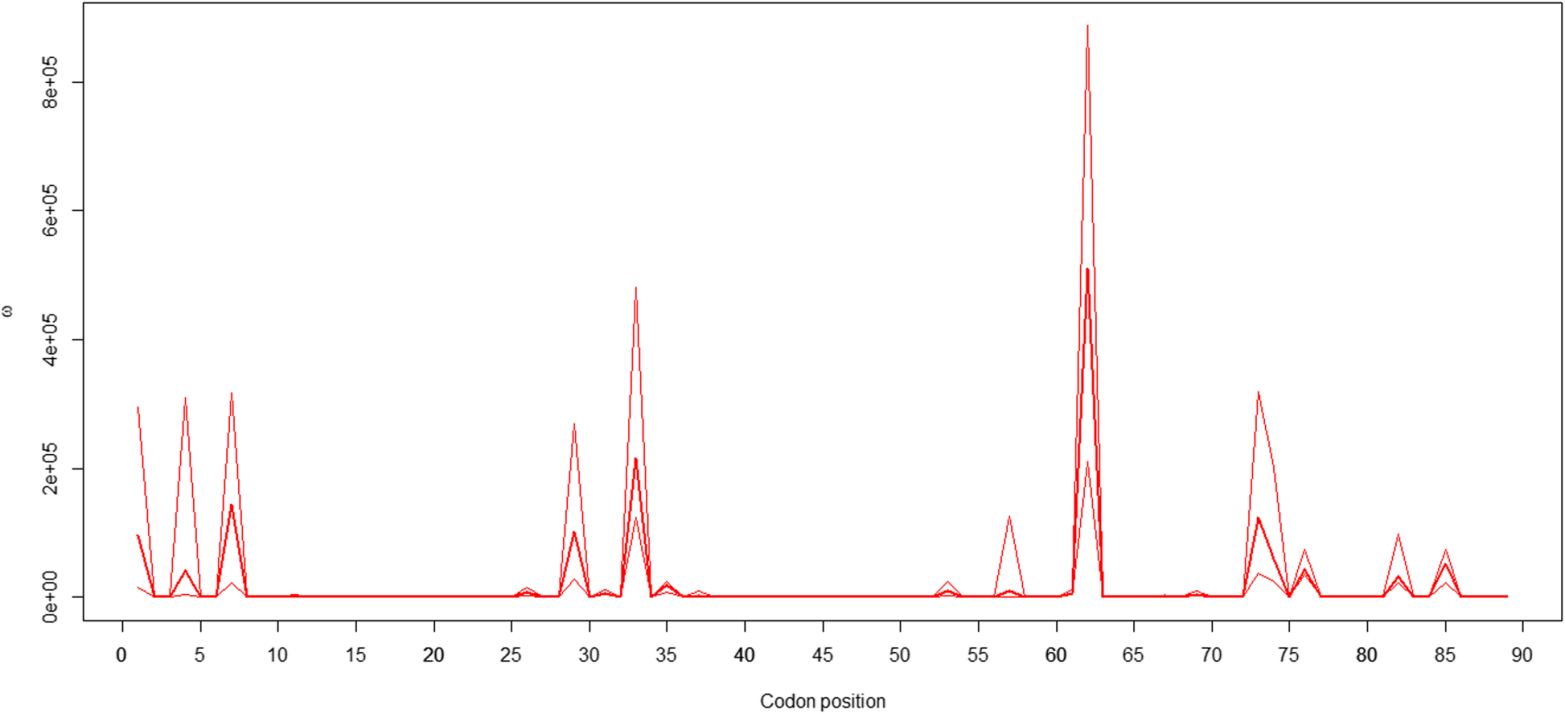
Estimated values of the selection index ω in each amino acid site along *Ovar-DRB1* exon 2 in Sudan Desert Sheep breeds: Abrag, Ashgar, Buze´e, Hamari, Kabashi, and Watish. Arrows indicate the antigen-binding site (ABS)

As shown in Table 3, na ranged from 17 in B to 33 in H breeds, while he and ho were higher than 0.88 and 0.82, respectively, in all populations; which evidence of the high diversity values for SDS. It is widely accepted that the genetic diversity of MHC class II genes can be maintained by balancing or overdominance selection. For this reason, HWE and Slatkin’s exact neutrality test were carried out to evaluate this phenomenon in SDS populations. However, the HWE test showed that genotype frequencies did not significantly deviate from the theoretical proportion. Furthermore, Slatkin’s exact neutrality test has only evidence of signature of selection compatible with balancing selection in B despite the even gene frequency profile mentioned above (Table 2). The obtained p value in B (*p* = 0.014) was consistent with the theoretical proportion expected under balancing pressure toward several alleles with low gene frequencies. In addition, we estimated the selection index (ω) in each amino acid site to evaluate the presence of diversifying selection (ω > 1) along *Ovar-DRB1* exon 2. These analyses showed high ω values in eleven sites in SDS, mainly located in the ABS (Fig. 5).

### *Ovar-DRB1 *genetic structure and levels of population differentiation in Sudan Desert Sheep

**Fig. 6:**
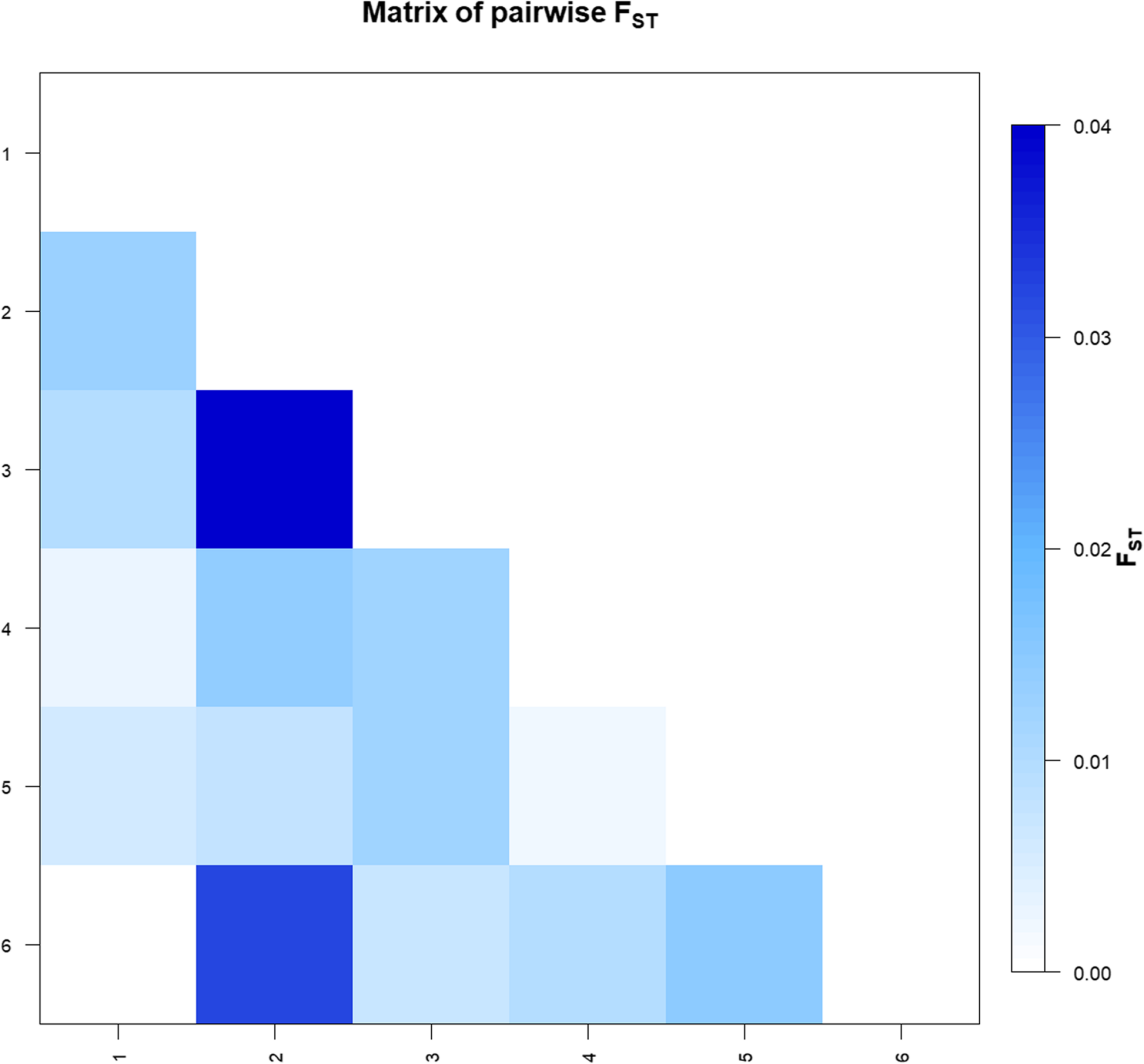
Graphic representation of calculated FST between population pairs using an R package pairFstMatrix.r. 1 = Abrag, 2 = Ashgar, 3 = Buze´e, 4 = Hamari, 5 = Kabashi, and 6 = Watish

**Fig. 7:**
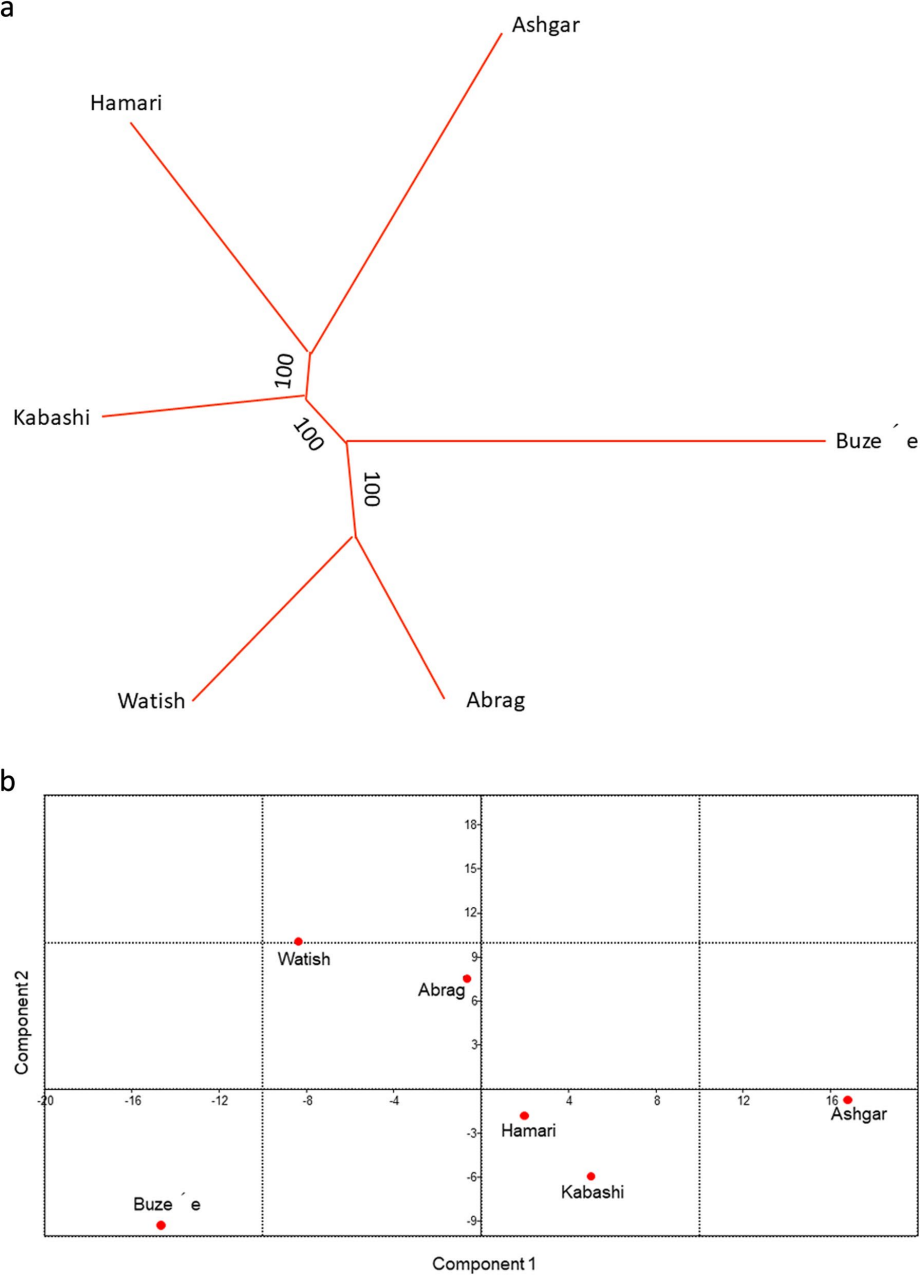
(**a**) Neighbor-joining dendrogram constructed from a matrix of DA genetic distances. (**b**) Principal Component Analysis of allele frequencies from the *Ovar-DRB1* gene in Sudan Desert Sheep breeds: Abrag, Ashgar, Buze´e, Hamari, Kabashi, and Watish

The average FST analysis showed a low but significant level of genetic differentiation for *Ovar-DRB1* gene between SDS breeds (FST = 0.010, *p* < 0.01), and the pairwise FST values varied between 0 (AB—W) to 0.040 (AS—B) (Table S1 and Fig. 6). Significant differences were observed in fourteen out of the fifteen comparisons (*p* < 0.05). In addtion, *Ovar-DRB1* allele frequencies from SDS were used to generate Nei’s DA and DS genetic distance matrices. Then, dendrograms were constructed from these distance matrices using UPGMA and NJ algorithms. All trees revealed congruent topologies, which were consistent with the historical and geographical location of the breeds, revealing two clusters which one included the easter breeds (AB, and W) and the other cluster comprised the western ones (AS, K and H), whereas B crossbreed located in intermediate position and AS diverge from the others breeds (Fig. 7a). In addition, *Ovar-DRB1* allele frequencies were used to perform PCA analyses among SDS breeds. The first PC accounted for 51.00% of the data variability, and clearly exhibited a differentiation pattern between eastern (negative values) and western (positive values) breeds, while AS and B were located in opposite extreme positions of the plot (Fig. 7b). The first PC was primarily determined by the contribution of negative values of the Ovar-DRB1*03:03, *03:07, *04:02, *12:02, *13:03, *14:02, and *16:08 alleles, and the positive values of Ovar-DRB1*01:01, *03:11, *06:03, *08:03, *10:01, and *Sudan1. The second PC explained 24.36% of the total variation and contributed to differentiate western and eastern SDS breeds. Finally, the third PC accounted for 11.73% of the variance, discriminating the populations within each cluster. In conclusion, the PCA results agree with the overall clustering observed after NJ or UPGMA tree construction.

In addition, PCA using the gene frequencies of the amino acid motif of the five pockets (pocket 1, pocket 4, pocket 6, pocket 7, and pocket 9) involved in the antigen-binding function of the MHC complex were carried out. In general, the same previously described spatial distributions were obtained, being the exception the pocket 4 (Fig. S4a-e). In addition, the B crossbred population presents a divergence position than other breeds in the PCA plots based on ABS motifs frequencies. PC1 explained from 40.75 to 90.53 of the total variance, while PC2 accounted for 5.41 to 34.94. Figures S4a-e showed the amino acid motifs that mainly contribute to the breed location in these PCAs.

## Discussion

The MHC Class II genes play a critical role in the immune response by presenting antigens to T-cells, thereby initiating the adaptive immune response [33]. This process is pivotal for effective pathogen recognition and the subsequent generation of immune responses tailored to combat a wide range of infectious agents [34]. Genetic diversity within MHC II genes, including *Ovar-DRB1* in sheep, is particularly important as it directly influences the repertoire of antigens that can be presented to T-cells, enabling a more comprehensive immune response [2, 35, 36]. In sheep, MHC II gene variation has been associated with differences in immune responsiveness and disease susceptibility. Studies have demonstrated that allelic polymorphisms in MHC II genes are linked to variations in immune responses against specific pathogens, including bacteria, viruses, and parasites [37, 38]. The diversity of MHC II alleles contributes to the sheep's ability to recognize and respond to a wide array of pathogens, thereby enhancing disease resistance [39]. Therefore, the breed specific variants identified genetic variation within the *Ovar-DRB1* gene significantly impacts immune system function in SDS. Allelic polymorphisms within *Ovar-DRB1* affect antigen-MHC II binding, influencing the specificity and efficiency of immune responses [40, 41]. Specific *Ovar-DRB1* alleles have been linked to differential resistance or susceptibility to diseases in sheep populations [42, 43], demonstrating the direct link between genetic diversity and immune response efficacy. The identification of breed-specific allelic diversity in the *Ovar-DRB1* gene among SDS breeds holds significant implications for disease resilience and adaptation. MHC II genetic variations contribute to unique immune response profiles in different breeds [44]. Breed-specific MHC II alleles have been associated with enhanced disease resistance in various livestock species [45, 46]. The breed-specific variants identified in SDS breeds may confer differential resistance or susceptibility to regional diseases, shedding light on how these breeds have evolved to cope with specific challenges.

Comparative analysis of *Ovar-DRB1* alleles in SDS with other sheep populations provides insights into genetic diversity and evolution. MHC II genes often exhibit population-specific allelic distributions due to ecological conditions and selection pressures [44]. Differences in prevalence and distribution of specific MHC II alleles have been documented in different geographic regions [47], suggesting local adaptation influences genetic diversity. Comparisons with other populations enhance understanding of allele distribution, genetic adaptation, and disease resistance strategies. The mean number of differences exceeded 16.73 (NPD range = 14.95 – 19.95) between all pairs of haplotypes in the sample serves as an estimate for the number of mutations that have occurred since the divergence of the haplotypes. This metric is instrumental in gauging genetic variation within populations. Elevated values signify increased genetic diversity, a factor that, in the context of DRB, may facilitate a response to a broad spectrum of pathogens. In comparison to other studies, the diversity observed in Ovar-DRB1 alleles aligns with that in other indigenous breeds [8, 48, 49] but surpasses that in commercially raised breeds within more controlled environments. This disparity can be attributed to natural selection, such as balancing selection in the case of DRB genes, recent gene migration between populations, and stochastic errors introduced by random genetic drift.

The diverse array of *Ovar-DRB1* alleles in SDS breeds has implications for genetic conservation and breeding. Understanding immune-related gene diversity is pivotal for safeguarding livestock populations [50]. Breed-specific variants may hold adaptive advantages specific to local environments or diseases, making them valuable candidates for conservation efforts [51]. Breeders can strategically incorporate specific alleles associated with resistance to enhance disease resistance and livestock performance [52]. Harnessing genetic diversity within *Ovar-DRB1* optimizes livestock health and productivity, contributing to sustainable and resilient production systems.

The exploration of *Ovar-DRB1* diversity in SDS holds implications for enhancing livestock health and resilience. Genetic diversity within immune genes enhances the ability to combat diseases and adapt to changing environments [53]. Breed-specific alleles may offer advantages in adapting to environmental stressors, contributing to robustness [54]. The study's findings emphasize the importance of informed breeding and management practices, contributing to more sustainable and resilient livestock production systems. The study highlights the value of cross-disciplinary collaboration in understanding genetic diversity's impact on health outcomes. Geneticists, immunologists, and livestock managers contribute synergistic insights [55]. Collaboration informs breeding strategies and promotes livestock health, resilience, and sustainability.

While this study has provided valuable insights into the genetic diversity of the *Ovar-DRB1* gene in SDS, there are certain limitations that warrant consideration. The sample size, although representative of multiple SDS breeds, may not fully encompass the broader genetic diversity present in these populations. Expanding the sample size to include additional individuals and breeds could provide a more comprehensive view of allelic variation [50]. Another limitation lies in the focus on the second exon of the *Ovar-DRB1* gene (Ovar-DRB1.2). Future studies could explore the entirety of the gene or even extend the analysis to other immune-related genes to unravel a more complete picture of the genetic determinants shaping immune responses in SDS [56, 57]. Additionally, the present study has laid the groundwork for understanding allelic diversity, but functional investigations of these alleles are essential. Investigating the associations between specific alleles and immune responses against different pathogens could offer deeper insights into the practical implications of genetic variation [34]. The significance of the four new alleles reported here lies in the fact that functional MHC DRB genes constitute one of the most polymorphic loci in the mammalian genome. The polymorphisms within these genes play a central role in the immune response to pathogens, as the efficiency of binding to antigenic peptides depends on the amino acid motifs present in the pockets of DRB molecules, specifically in exon 2. According to the IPD-MHC database, 130 Ovar-DRB1 alleles have been reported, but their geographic and breed distribution is uneven. Some alleles are widely distributed across different regions and breeds, while others are detected solely in specific regions or closely related breed groups. Indigenous breeds serve as a crucial source of DRB genetic diversity. The geographic distribution of these alleles is a legacy of historical, stochastic (genetic drift), or selective (natural or artificial) factors. Conducting functional or association studies in Sudan Desert Sheep is essential to comprehend the contribution of these newly identified alleles.

Future directions for research could encompass longitudinal studies to monitor changes in allele frequencies over time and in response to evolving disease pressures. Integrating genomic data with phenotypic and environmental data would allow for a more comprehensive assessment of genotype–phenotype interactions and adaptive mechanisms within SDS populations.

## Conclusion

The comprehensive exploration of allelic diversity within the *Ovar-DRB1* gene in SDS has provided valuable insights into the genetic foundations of immune responses and disease resistance. Our findings underscore the intricate interplay between genetics and health outcomes in livestock populations. The identification of breed-specific variants and novel alleles within *Ovar-DRB1* highlights the unique adaptive potential of different SDS breeds. These insights have direct implications for informed breeding strategies, disease management, and conservation efforts. By unraveling the complex relationships between genetic diversity and immune responses, this study contributes to the advancement of livestock health and resilience.

## Future perspectives

As we move forward, several avenues for future research emerge from this study. Expanding the scope of genetic analysis beyond the *Ovar-DRB1* gene to include other immune-related genes could provide a more comprehensive understanding of immune system dynamics in SDS populations. Longitudinal studies tracking allele frequencies over time and in response to changing disease pressures will shed light on the evolving genetic landscape and adaptive mechanisms. Integrating genomic data with phenotypic and environmental information will enable a holistic assessment of genotype–phenotype interactions. Collaborative efforts among geneticists, immunologists, livestock managers, and policy makers remain essential. Such interdisciplinary cooperation will facilitate the translation of genetic insights into practical strategies for enhancing livestock health, productivity, and resilience. Additionally, continued collaboration with international partners will enable cross-comparisons with other sheep populations, further enriching our understanding of genetic diversity and adaptation. In conclusion, this study serves as a stepping stone towards harnessing genetic diversity for the betterment of Sudan Desert Sheep populations. By addressing limitations, embracing cross-disciplinary collaboration, and embarking on future research endeavors, we can unlock the full potential of genetic diversity to ensure the health and prosperity of these valuable livestock breeds.

### Supplementary Information

**Additional file 1. Fig S1. **Neighbor-joining (NJ) tree constructed from the nucleotide sequence that only encoded the antigen-binding site (ABS) by all reported *Ovar-DRB1* alleles and the four new ones (From Ovar-DRB1*Sudan1 to Ovar-DRB1*Sudan4) detected in the Sudan desert sheep breed. Numbers are bootstrap percentages that support each node. Bootstrapping was carried up with 1000 replicates to assess the reliability of individual branches. Abrag (AB), Ashgar (AS), Buze´e, Hamari (H), Kabashi (K), and Watish (W). Breeds where alleles were detected are indicated between brackets.

**Additional file 2. Fig. S2. **Cumulative gene frequency plot of *Ovar-DRB1* alleles in the Sudan Desert  sheep breed: Abrag (AB), Ashgar (AS), Buze´e, Hamari (H), Kabashi (K), and Watish (W).

**Additional file 3. Fig S3. **Venn plot of the common Ovar-DRB1 alleles shared by Sudan Desert Sheep breeds: Abrag, Ashgar, Hamari, Kabashi, and Watish breeds.

**Additional file 4. Fig. S4. **a-e. Principal component analysis of *Ovar-DRB1* gene using the pocket amino acid motifs frequencies in Sudan Desert Sheep (Abrag, Ashgar, Buze´e, Hamari, Kabashi, and Watish): a. Pocket 1, b. Pocket 4, c. Pocket 6, d. Pocket 7, and e. Pocket 9. Percentage of the total variance accounted for the first principal components (PC1, PC2, and PC3) were detailed.

**Additional file 5. Table S1. **Genetic distance between pairs of breeds/populations estimated through Nei DA distance (above) and F_ST_ (below; *p* values in parentheses) in Sudan Desert Sheep breeds: Abrag (AB), Ashgar (AS), Buze´e (B), Hamari (H), Kabashi (K), and Watish (W).

## Data Availability

The Illumina MiSeq sequences obtained were deposited to the DNA Data Bank of Japan (https://www.ncbi.nlm.nih.gov/sra/?term =) under accession number DRA017273. The processed sequences of the all detected alleles in the SDS were submitted to Genbank under accession numbers OR488718-OR488752, OR488756, OR488764, OR488770, and OR636395 (https://www.ncbi.nlm.nih.gov/Genbank/update.html).
