# Supplementary figures and images for: Exploring genetic diversity and variation of Ovar-DRB1 gene in Sudan Desert Sheep using targeted next-generation sequencing
Source: BMC Genomics. 2024 Feb 8;25:160. doi: 10.1186/s12864-024-10053-3 (PMC10851530; doi:10.1186/s12864-024-10053-3)

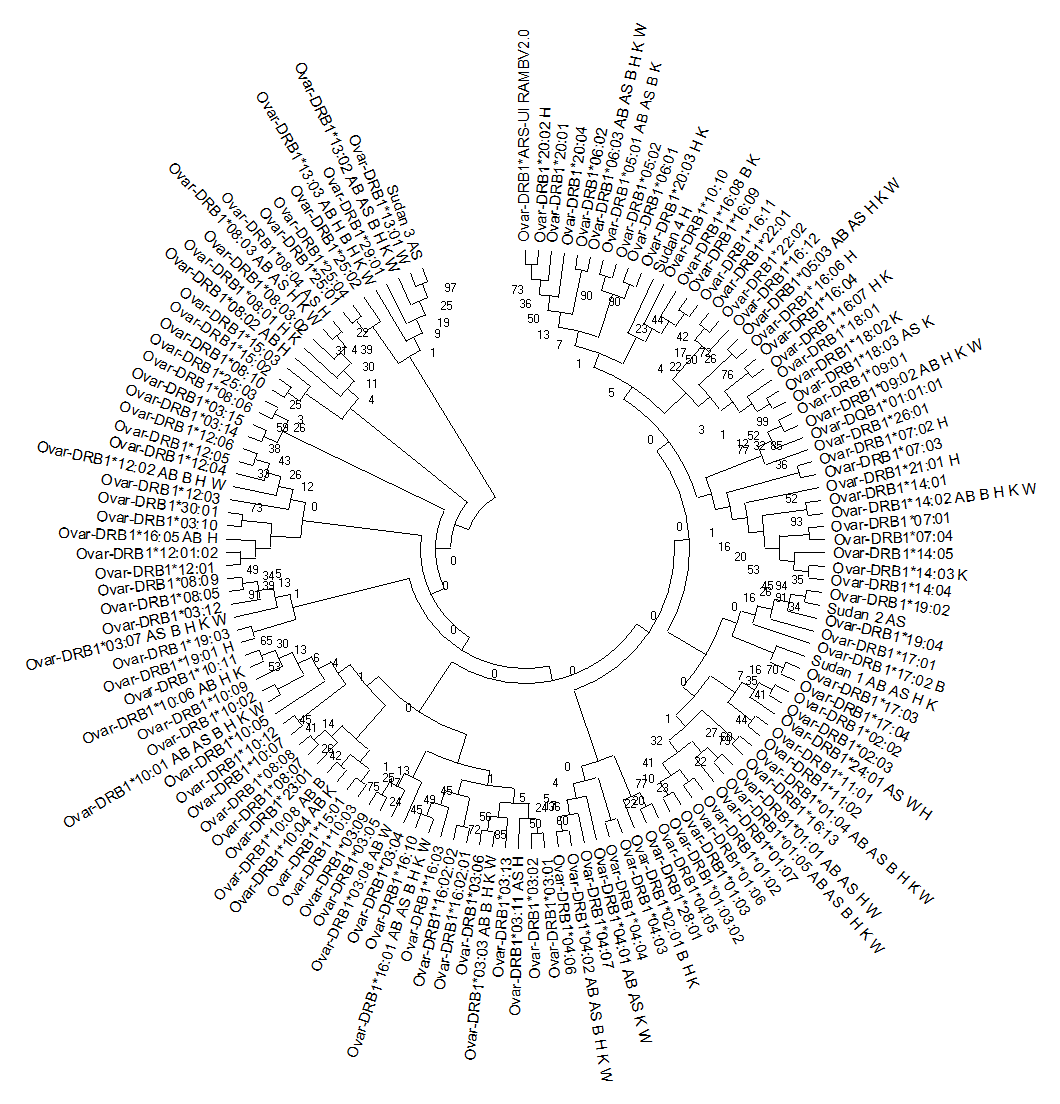

Supplement: Supplementary file 1 — Additional file 1. Fig S1. Neighbor-joining (NJ) tree constructed from the nucleotide sequence that only encoded the antigen-binding site (ABS) by all reported Ovar-DRB1 alleles and the four new ones (From Ovar-DRB1*Sudan1 to Ovar-DRB1*Sudan4) detected in the Sudan desert sheep breed. Numbers are bootstrap percentages that support each node. Bootstrapping was carried up with 1000 replicates to assess the reliability of individual branches. Abrag (AB), Ashgar (AS), Buze´e, Hamari (H), Kabashi (K), and Watish (W). Breeds where alleles were detected are indicated between brackets. [file 12864_2024_10053_MOESM1_ESM.tif]

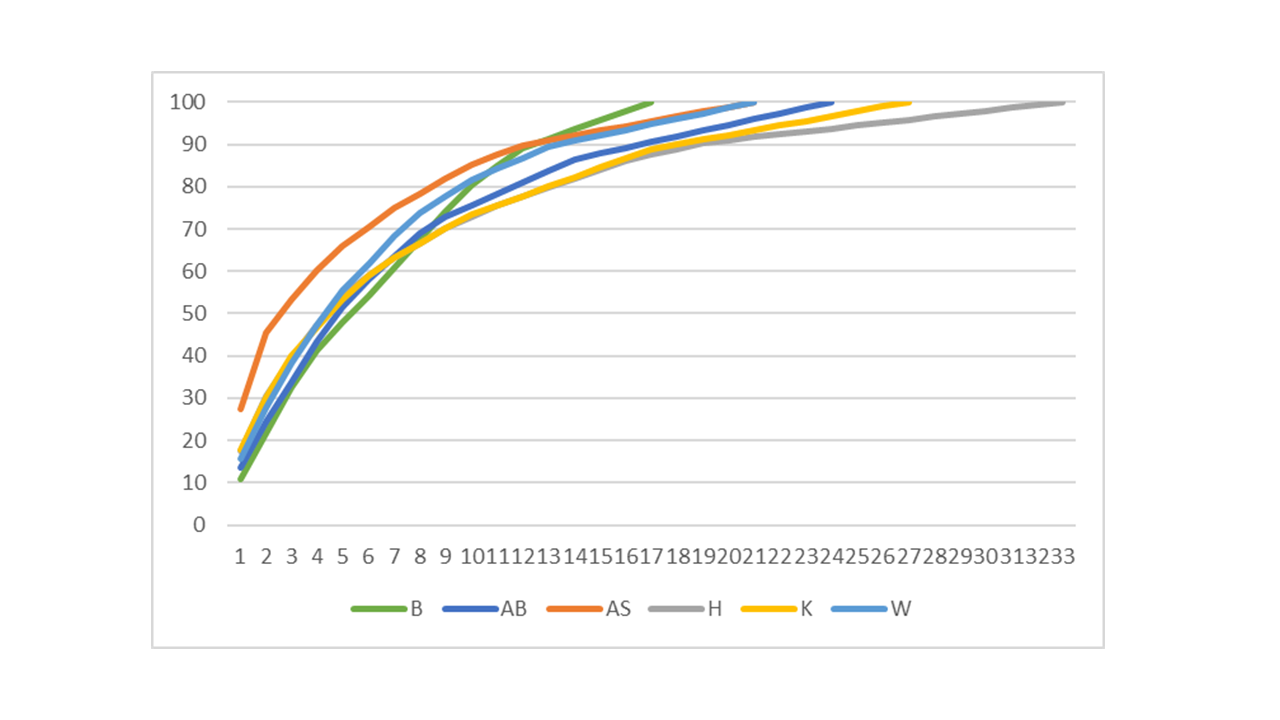

Supplement: Supplementary file 2 — Additional file 2. Fig. S2. Cumulative gene frequency plot of Ovar-DRB1 alleles in the Sudan Desert sheep breed: Abrag (AB), Ashgar (AS), Buze´e, Hamari (H), Kabashi (K), and Watish (W). [file 12864_2024_10053_MOESM2_ESM.tif]

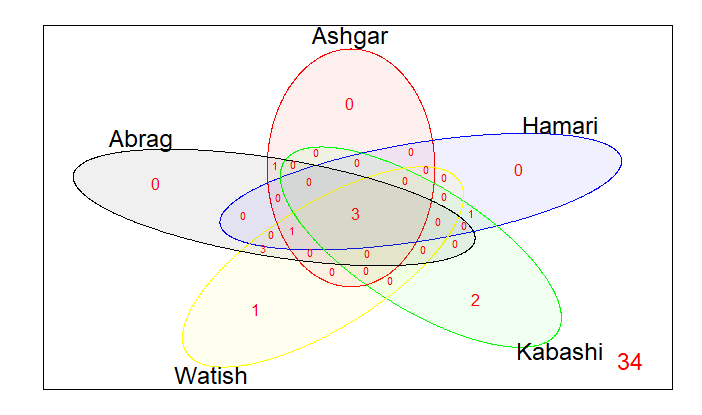

Supplement: Supplementary file 3 — Additional file 3. Fig S3. Venn plot of the common Ovar-DRB1 alleles shared by Sudan Desert Sheep breeds: Abrag, Ashgar, Hamari, Kabashi, and Watish breeds. [file 12864_2024_10053_MOESM3_ESM.tif]

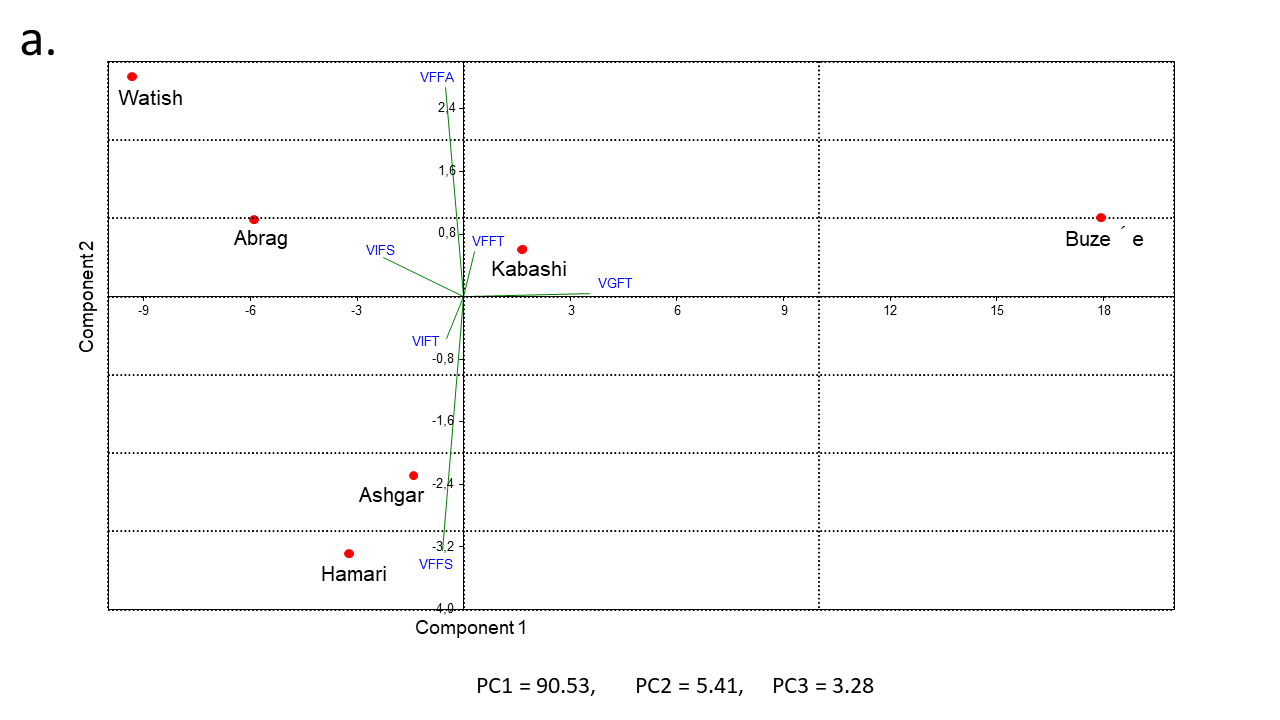

Supplement: Supplementary file 4 — Additional file 4. Fig. S4. a-e. Principal component analysis of Ovar-DRB1 gene using the pocket amino acid motifs frequencies in Sudan Desert Sheep (Abrag, Ashgar, Buze´e, Hamari, Kabashi, and Watish): a. Pocket 1, b. Pocket 4, c. Pocket 6, d. Pocket 7, and e. Pocket 9. Percentage of the total variance accounted for the first principal components (PC1, PC2, and PC3) were detailed. [file 12864_2024_10053_MOESM4_ESM.zip › fig. s4/Figure S4a_PCA motif pocket 1.tif]

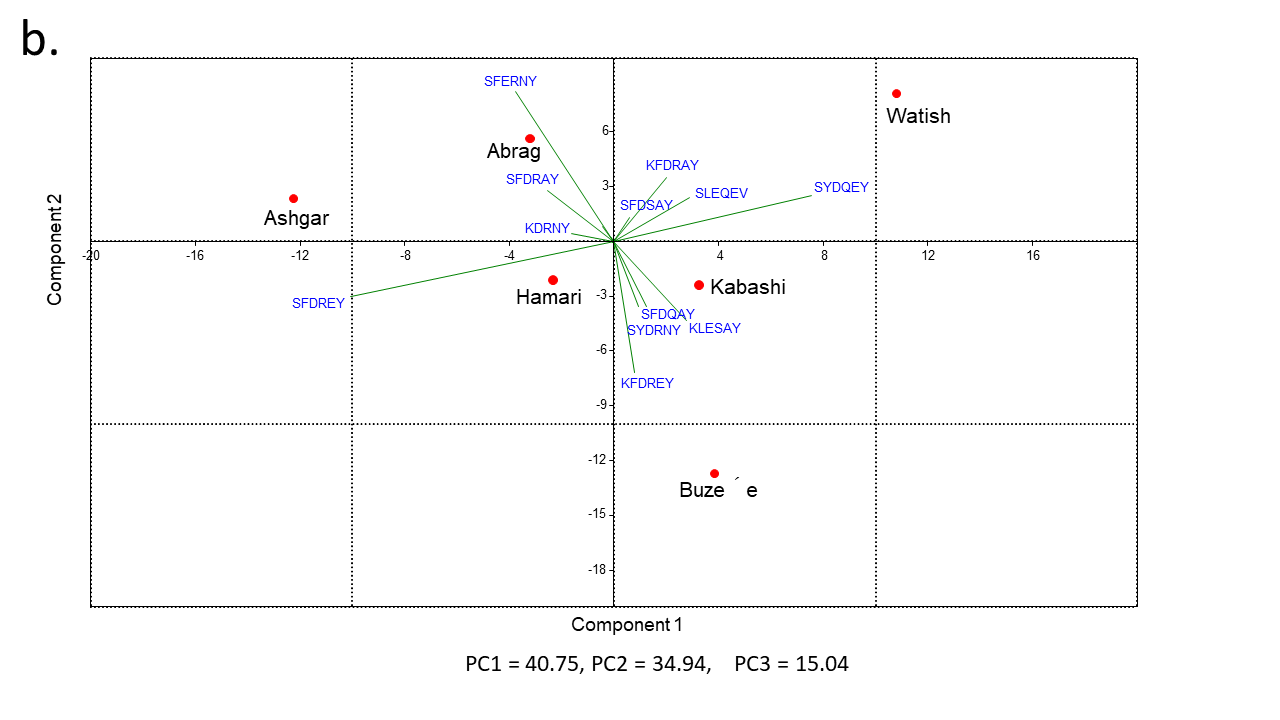

Supplement: Supplementary file 4 — Additional file 4. Fig. S4. a-e. Principal component analysis of Ovar-DRB1 gene using the pocket amino acid motifs frequencies in Sudan Desert Sheep (Abrag, Ashgar, Buze´e, Hamari, Kabashi, and Watish): a. Pocket 1, b. Pocket 4, c. Pocket 6, d. Pocket 7, and e. Pocket 9. Percentage of the total variance accounted for the first principal components (PC1, PC2, and PC3) were detailed. [file 12864_2024_10053_MOESM4_ESM.zip › fig. s4/Figure S4a_PCA motif pocket 4.tif]

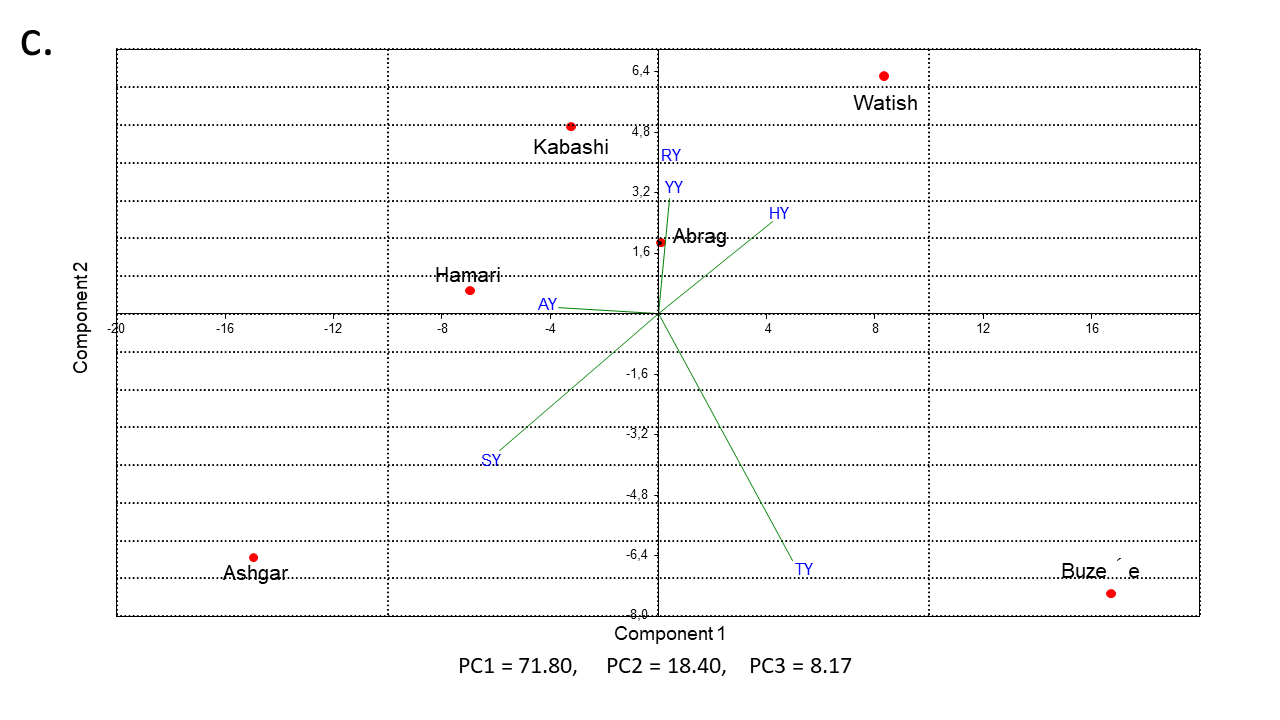

Supplement: Supplementary file 4 — Additional file 4. Fig. S4. a-e. Principal component analysis of Ovar-DRB1 gene using the pocket amino acid motifs frequencies in Sudan Desert Sheep (Abrag, Ashgar, Buze´e, Hamari, Kabashi, and Watish): a. Pocket 1, b. Pocket 4, c. Pocket 6, d. Pocket 7, and e. Pocket 9. Percentage of the total variance accounted for the first principal components (PC1, PC2, and PC3) were detailed. [file 12864_2024_10053_MOESM4_ESM.zip › fig. s4/Figure S4a_PCA motif pocket 6.tif]

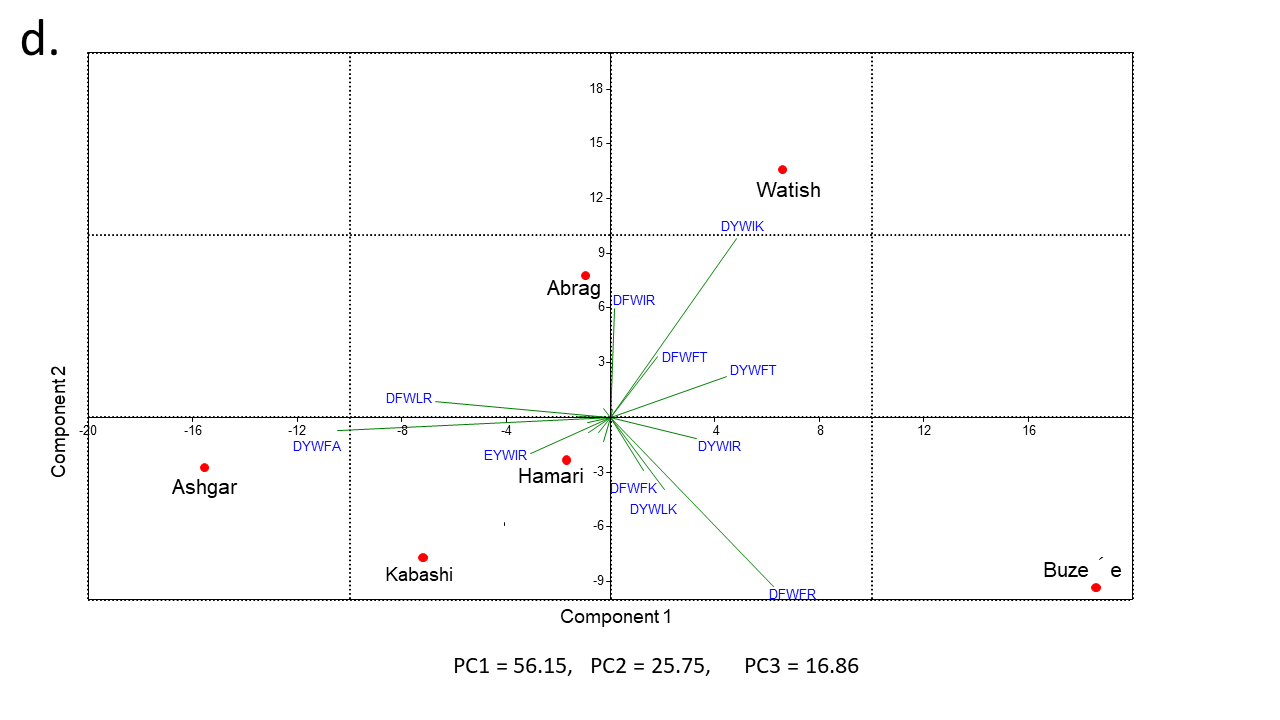

Supplement: Supplementary file 4 — Additional file 4. Fig. S4. a-e. Principal component analysis of Ovar-DRB1 gene using the pocket amino acid motifs frequencies in Sudan Desert Sheep (Abrag, Ashgar, Buze´e, Hamari, Kabashi, and Watish): a. Pocket 1, b. Pocket 4, c. Pocket 6, d. Pocket 7, and e. Pocket 9. Percentage of the total variance accounted for the first principal components (PC1, PC2, and PC3) were detailed. [file 12864_2024_10053_MOESM4_ESM.zip › fig. s4/Figure S4a_PCA motif pocket 7.tif]

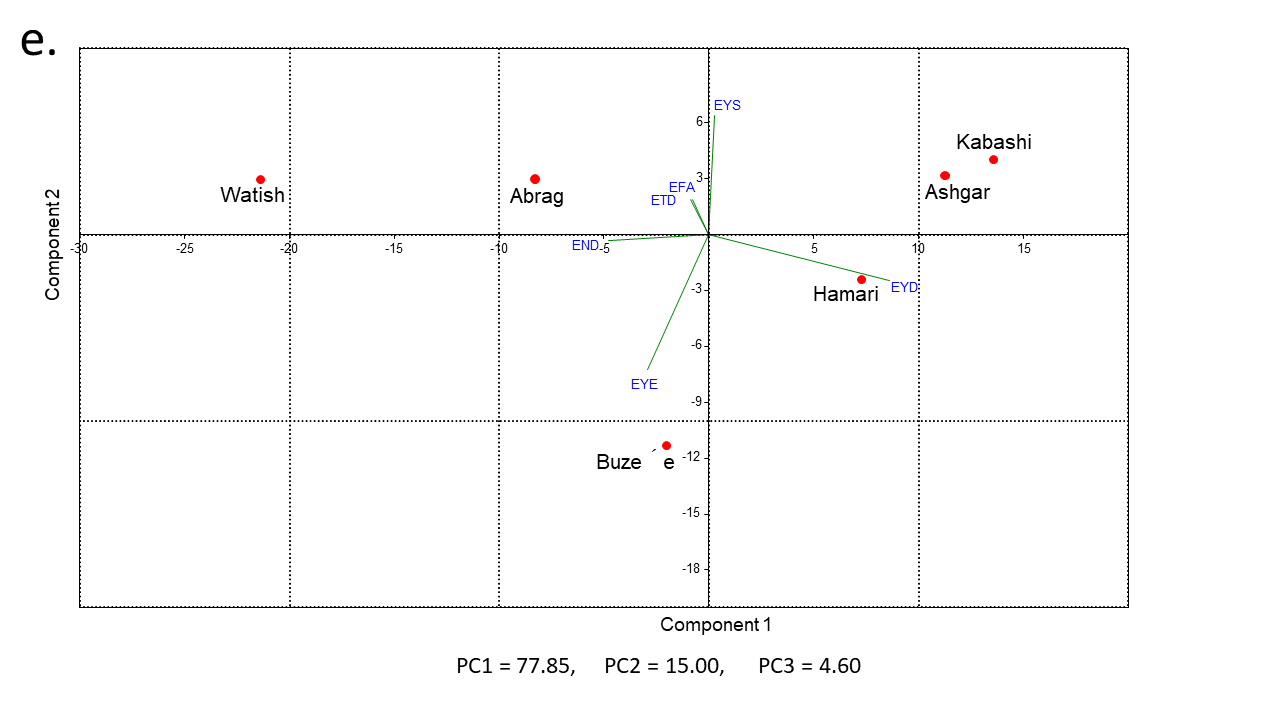

Supplement: Supplementary file 4 — Additional file 4. Fig. S4. a-e. Principal component analysis of Ovar-DRB1 gene using the pocket amino acid motifs frequencies in Sudan Desert Sheep (Abrag, Ashgar, Buze´e, Hamari, Kabashi, and Watish): a. Pocket 1, b. Pocket 4, c. Pocket 6, d. Pocket 7, and e. Pocket 9. Percentage of the total variance accounted for the first principal components (PC1, PC2, and PC3) were detailed. [file 12864_2024_10053_MOESM4_ESM.zip › fig. s4/Figure S4a_PCA motif pocket 9.tif]
