# Supplementary material for: Exploring genetic diversity and variation of Ovar-DRB1 gene in Sudan Desert Sheep using targeted next-generation sequencing
Source: BMC Genomics. 2024 Feb 8;25:160. doi: 10.1186/s12864-024-10053-3 (PMC10851530; doi:10.1186/s12864-024-10053-3)
Supplement: Supplementary file 5 — Additional file 5. Table S1. Genetic distance between pairs of breeds/populations estimated through Nei DA distance (above) and FST (below; p values in parentheses) in Sudan Desert Sheep breeds: Abrag (AB), Ashgar (AS), Buze´e (B), Hamari (H), Kabashi (K), and Watish (W). [file 12864_2024_10053_MOESM5_ESM.docx]

**Table S1.** Genetic distance between pairs of breeds/populations estimated through Nei DA distance (above) and F_ST_ (below; p values in parentheses) in Sudan Desert Sheep breeds: Abrag (AB), Ashgar (AS), Buze´e (B), Hamari (H), Kabashi (K), and Watish (W).

| **Breed** | **AB** | **AS** | **B** | **H** | **K** | **W** |
| --- | --- | --- | --- | --- | --- | --- |
| **AB** | 0 | 0.232 | 0.317 | 0.201 | 0.275 | 0.154 |
| **AS** | 0.013 (0.016) | 0 | 0.392 | 0.227 | 0.248 | 0.279 |
| **B** | 0.010 (0.010) | 0.040 (< 0.001) | 0 | 0.271 | 0.296 | 0.264 |
| **H** | 0.003 (0.040) | 0.014 (0.002) | 0.012 (0.007) | 0 | 0.201 | 0.229 |
| **K** | 0.006 (0.008) | 0.008 (0.006) | 0.012 (0.012) | 0.002 (0.019) | 0 | 0.302 |
| **W** | -0.005 (0.701) | 0.032 (< 0.001) | 0.007 (0.011) | 0.010 (0.002) | 0.015 (< 0.001) | 0 |
